# Susceptibility profile and metabolic mechanisms involved in *Aedes aegypti* and *Aedes albopictus* resistant to DDT and deltamethrin in the Central African Republic

**DOI:** 10.1186/s13071-016-1887-5

**Published:** 2016-11-24

**Authors:** Carine Ngoagouni, Basile Kamgang, Cécile Brengues, Gildas Yahouedo, Christophe Paupy, Emmanuel Nakouné, Mirdad Kazanji, Fabrice Chandre

**Affiliations:** 1 Institut Pasteur de Bangui, PO Box 923, Bangui, Central African Republic; 2 Research Unit Liverpool School of Tropical Medicine, Organisation de Coordination pour la lutte contre les Endémies en Afrique Centrale, PO Box 288, Yaoundé, Cameroon; 3 Laboratoire des Maladies Infectieuses et Vecteurs: Ecologie, Génétique, Evolution et Contrôle, Unité mixte de Recherche 224–5290, Centre National de Recherche Scientifique-Institut de Recherche pour le Développement, Université de Montpellier, Montpellier, France; 4 Institut Pasteur de la Guyane, BP 6010, 23 Avenue Pasteur, 97306 Cayenne, French Guiana

**Keywords:** *Aedes aegypti*, *Aedes albopictus*, Arboviruses, Vectors, Insecticide resistance, Metabolic resistance, Central African Republic

## Abstract

**Background:**

*Aedes aegypti* and *Ae. albopictus* are the main epidemic vectors of dengue, chikungunya and Zika viruses worldwide. Their control during epidemics relies mainly on control of larvae and adults with insecticides. Unfortunately, loss of susceptibility of both species to several insecticide classes limits the efficacy of interventions. In Africa, where *Aedes*-borne viruses are of growing concern, few data are available on resistance to insecticides. To fill this gap, we assessed the susceptibility to insecticides of *Ae. aegypti* and *Ae. albopictus* populations in the Central African Republic (CAR) and studied the mechanisms of resistance.

**Methods:**

Immature stages were sampled between June and September 2014 in six locations in Bangui (the capital of CAR) for larval and adult bioassays according to WHO standard procedures. We also characterized DDT- and pyrethroid-resistant mosquitoes molecularly and biochemically, including tests for the activities of nonspecific esterases (α and β), mixed-function oxidases, insensitive acetylcholinesterase and glutathione *S*-transferases.

**Results:**

Larval bioassays, carried out to determine the lethal concentrations (LC_50_ and LC_95_) and resistance ratios (RR_50_ and RR_95_), suggested that both vector species were susceptible to *Bacillus thuringiensis* var*. israeliensis* and to temephos. Bioassays of adults showed susceptibility to propoxur and fenitrothion, except for one *Ae. albopictus* population that was suspected to be resistant to fenithrothion. None of the *Ae. aegypti* populations was fully susceptible to DDT. *Ae. albopictus* presented a similar profile to *Ae. aegypti* but with a lower mortality rate (41%). Possible resistance to deltamethrin was observed among *Ae. aegypti* and *Ae. albopictus*, although some were susceptible. No *kdr* mutations were detected in either species; however, the activity of detoxifying enzymes was higher in most populations than in the susceptible *Ae. aegypti* strain, confirming decreased susceptibility to DDT and deltamethrin.

**Conclusion:**

These findings suggested that regular, continuous monitoring of resistance is necessary in order to select the most effective adulticides for arbovirus control in Bangui.

**Electronic supplementary material:**

The online version of this article (doi:10.1186/s13071-016-1887-5) contains supplementary material, which is available to authorized users.

## Background

*Aedes aegypti* and *Ae. albopictus* are the main vectors of dengue virus (DENV, *Flaviviridae*, *Flavivirus*), chikungunya virus (CHIKV, *Togaviridae*, *Alphavirus*) and Zika virus (ZIKV, *Flaviviridae*, *Flavivirus*) worldwide. *Aedes aegypti* is native to Africa and is found throughout the tropics, whereas *Ae. albopictus* is native to Asia but has recently spread to all continents. These three viruses are increasing global health threats, spreading from their original niches to most areas of the world. Dengue virus infection is a serious health problem for 2.5 billion people worldwide. A recent estimate indicated 390 million infections per year (95% credible interval, 284–528 million), of which 96 million (67–136 million) manifest clinically, although the actual number of cases is under-reported, and many cases are not definitively classified [1]. Dengue infection causes more illness and deaths in humans than any other arboviral disease, the severity ranging from mild dengue fever to dengue haemorrhagic fever and dengue shock syndrome. Chikungunya fever is also as a significant worldwide public health problem; particular attention has been paid to this disease after its rapid, massive emergence in the south-west Indian Ocean, India and the Americas [2]. Chikungunya can manifest as asymptomatic to severe infections, mainly with severe arthralgia but also with cardiovascular, neurological and respiratory symptoms [3, 4]. ZIKV, which was originally isolated in 1947 from a monkey in Uganda [5], has occasionally been reported in humans in Africa and Asia [6], but, after its sudden emergence in 2007 on Yap Island, affecting about 5,000 people [7], it caused successive outbreaks in French Polynesia in 2013–2014 [8] and in Brazil since October 2015, before spreading across Latin America [9]. The recrudescence of ZIKV has been associated with reports of neurological disorders and microcephaly, and the World Health Organization (WHO) declared it a “public health emergency of international concern” on 1 February 2016 [10].

In Central Africa, several arboviruses, including DENV, CHIKV and ZIKV have been isolated from mosquitoes and human samples, but no massive outbreak was reported before introduction of the new competent vector *Ae. albopictus*. After it was first reported in 2000 in Cameroon [11], it spread rapidly to numerous countries in Central Africa and, with the native species *Ae. aegypti*, played a role in disseminating and spreading DENV, CHIKV and ZIKV in urban and rural environments [6, 12–14]. This change in the epidemiology of these three arboviruses is particularly disturbing because the region has many potentially suitable niches for *Ae. albopictus*, and several arboviruses of medical and veterinary concern are endemic [15]. In view of the current lack of effective vaccines or specific drugs against these arboviral infections, vector control remains the most effective means for their prevention [16].

Insecticides play a major role in the control of mosquito vectors, and synthetic pyrethroids are the chemicals of choice because of their effective, rapid activity against insects, their low toxicity for mammals and their degradability in the environment [17, 18]. WHO recommends use of pyrethroids against adult mosquitoes and larvicides such as *Bacillus thuringiensis* var*. israeliensis* (*Bti*), organophosphates like temephos, benzoylureas like diflubenzuron, juvenile hormone mimics like pyriproxyfen and spinosyns such as spinosad for larval control [19]. Unfortunately, long-term intensive use of insecticides usually leads to the emergence of resistance in mosquito species under selection pressure, and this is one of the major obstacles to the control of arthropod pests [20, 21]. Many control programmes are threatened by insecticide resistance in *Ae. aegypti* and *Ae. albopictus. Aedes aegypti* has been reported to be resistant to pyrethroids and organophosphates in various part of the world, whereas few data are available on insecticide resistance in *Ae. albopictus*. The few studies that have been conducted show decreased susceptibility of *Ae. albopictus* to a wide variety of active ingredients, including organophosphates, organochlorines (DDT) and pyrethroids.

Insecticide resistance in insects is due to two main mechanisms: enhanced metabolic detoxification and insensitivity of target sites [22]. The first mechanism involves overexpression or qualitative changes in the catalytic sites of enzymes such as non-specific esterases (NES), glutathione *S*-transferases (GST) and mixed-function oxidases (MFO). Previous studies outside Africa on metabolic resistance mechanism in *Ae. aegypti* and *Ae. albopictus* showed that most of detoxification enzymes linked to insecticide resistance belong to the cytochrome P450 genes [23, 24]. Target insensitivity is due to mutations that reduce the binding affinity between the insecticide and its physiological target. Pyrethroids and DDT acts on voltage-sensitive sodium channels, and insects develop resistance to these types of insecticide through substitution(s) of one or several amino acids in the channel sequence [25]. These mutations in the voltage-sensitive sodium channel are known as “knockdown resistance” (*kdr*) and have been reported in several mosquito genera, including *Anopheles gambiae* [26], *An. stephensi* [27], *Culex pipiens* [28] and *Ae. aegypti* [29]. In many mosquito species, including *Ae. aegypti*, *kdr* related to pyrethroids and DDT has been located in segment 6 of domain II [23, 29–31], with other mutations at the same position (*I1011M*, *I1011V*, *V1016G* and *V1016I*) [32]. In South-East Asia, for example, two major voltage-gated sodium channel haplotypes (*S989P* + *V1016G* and *F1534C*) confer resistance of *Ae. aegypti* to pyrethroids, and species with these two haplotypes are widely and sympatrically distributed in that region [33–35]. Neurophysiological studies have revealed that *V1016G* and *F1534C* single mutations each confer resistance to pyrethroids [36]. Such *kdr* mutations are rare in *Ae. albopictus*; the mutation *F1534C* (TTC to TGC) at segment 6 of domain III described in *Ae. aegypti* is the only one that has been confirmed in *Ae. albopictus* from Singapore [37].

In Africa, most of the data on insecticide susceptibility concerns malaria vectors (*Anopheles* mosquitoes), and very little is known about *Aedes*. Most of the studies on *Ae. aegypti* in West and Central Africa date back 30–40 years [38], although resistance of *Ae. aegypti* and *Ae. albopictus* was reported in Cameroon after introduction of *Ae. albopictus* [39]. In CAR, both species are present, but *Ae. albopictus* predominates over *Ae. aegypti* at all sites in Bangui and southern CAR, where the two species are sympatric [40]. The lack of data on their susceptibility to insecticides used in public health is a growing obstacle for dengue, chikungunya and Zika disease control programmes. We assessed the susceptibility of larvae and adults of *Ae. aegypti* and *Ae. albopictus* to insecticides and the mechanism involved in order to select the best insecticides for use in an outbreak and to manage resistance in these populations.

## Methods

### Mosquito strains and collection

**Fig. 1:**
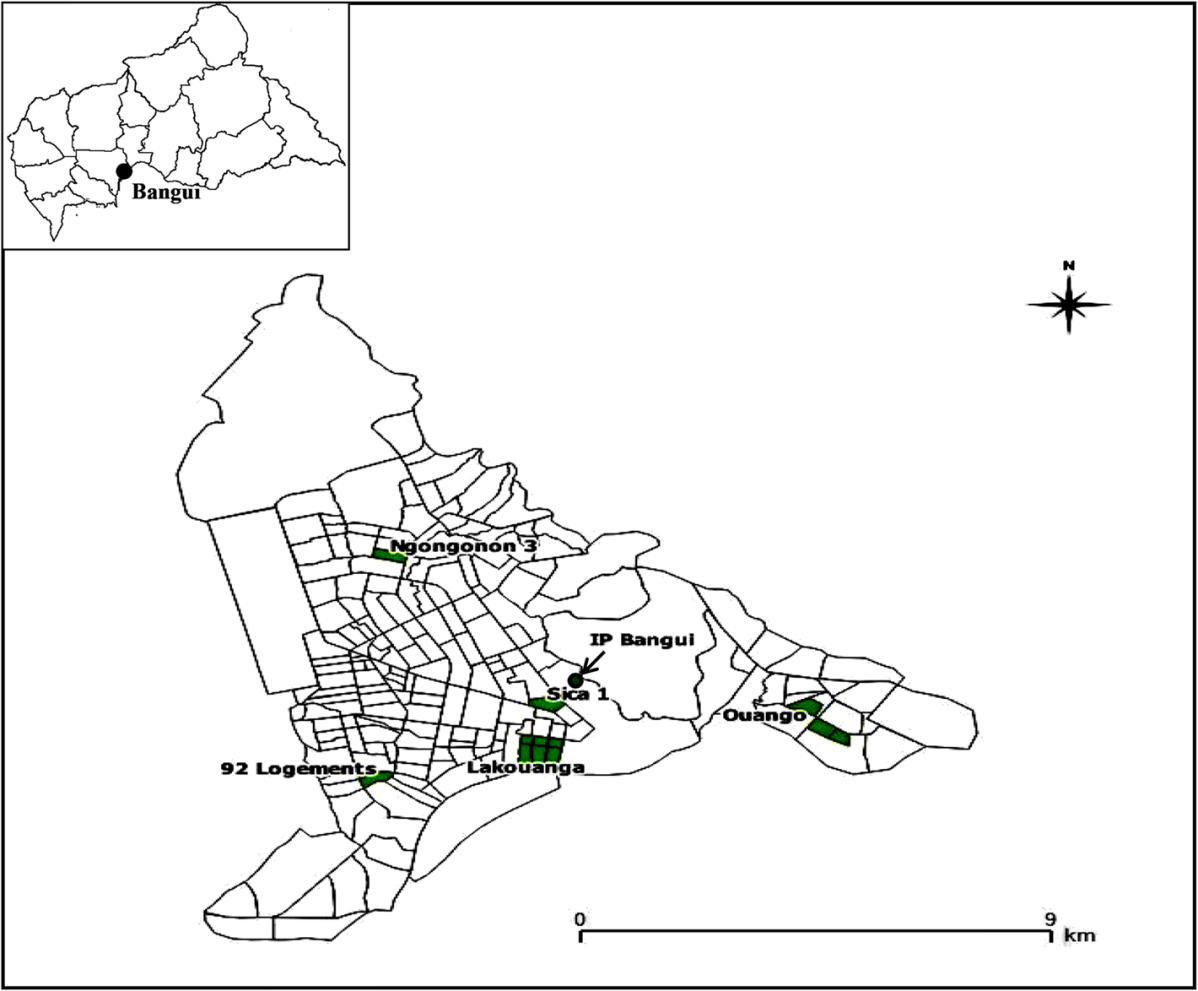
Map of Bangui showing sampling sites

Field-caught larval or pupal *Ae. aegypti* and *Ae. albopictus* (F_0_ generation) were sampled between June and September 2014, during the rainy season, in six districts of Bangui (Fig. 1). All samples were taken to the insectaries at the Institut Pasteur de Bangui (IPB) and maintained under controlled conditions (28 ± 2 °C and 80 ± 10% relative humidity); larvae were fed dry cat food. After emergence, adult *Aedes* mosquitoes were identified morphologically [41], grouped by species and site into cages and fed 10% sugar solution. Some of the F_0_ adults were used in adult bioassays, molecular and biochemical studies, and the remainder were used to obtain the next generation (F_1_). Females were allowed to feed on blood from rabbits to induce egg-laying. F_1_ eggs were hatched to obtain larvae for use in larval bioassays. The insecticides tested were chosen from the four main classes of adulticides (organochlorines, pyrethroids, carbamates and organophosphates) and two of larvicides (*Bti* and temephos). The reference strain (*Ae. aegypti* SBE) originating from Benin and papers impregnated with adulticides and *Bti* were supplied by the MiVEGEC project (Maladies Infectieuses et Vecteurs: Écologie, Génétique, Évolution, contrôle) (Université de Montpellier, France).

### Larval bioassays

Larval bioassays were performed according to standard WHO guidelines [42] using F1 larvae. The susceptibility of larvae was evaluated against technical-grade temephos (97.3%; Sigma Aldrich-Pestanal®, Seelze, Germany) and a formulated *Bti* product (Vectobac® 12AS, 1200 ITU/mg). First, stock solutions and serial dilutions were prepared in 95% ethanol (temephos) or distilled water (*Bti*) and stored at 4 °C. For *Bti*, five concentrations ranging from 0.08 to 0.2 mg/l and for temephos, eight doses ranging from 0.0025 to 0.009 mg/l have been used in larval bioassay. Fifty to 100 larvae per concentration (with three to four replicates, depending on the sample and the number of larvae available) were tested. Third late or early fourth-instar larvae of each species were placed in plastic cups containing 99 ml of tap water, and 1 ml of insecticide solution at the required concentration was added. Control groups were run systematically with larvae exposed to 1 ml of ethanol (for temephos) or 1 ml of tap water (for *Bti*). No food was provided to larvae during the bioassays, which were run at 28 ± 2 °C and 80 ± 10% relative humidity. Mortality was determined after 24 h of exposure to the insecticide. Mortality rates were corrected with Abbott’s formula [43] when the mortality of controls was > 5%.

All data were analysed with Win DL v. 2.0 software [44]. Lethal concentrations (LC_50_ and LC_95_) were calculated with their 95% confidence intervals (CIs). Resistance ratios (RR_50_ and RR_95_) were calculated by comparing the LC_50_ and LC_95_ for each species with those of susceptible strain, as RR_50(95)_ = LC_50(95)_ of studied population/LC_50(95)_ susceptible strain and RR_95_ = LC_95(95)_ of studied population/LC_95(95)_ reference strain. Mosquito populations were considered as resistant when RR was higher than 1 and confidence intervals for LC_50_ or LC_95_ did not overlap those of the susceptible reference strain.

### Adult bioassays

Adult bioassays were performed with non-blood-fed females according to the standard WHO guidelines [42]. Four insecticides 4% DDT (organochlorine), 0.05% deltamethrin (pyrethroid), 0.1% propoxur (carbamate) and 0.5% fenitrothion (organophosphate) were tested. Two to four batches of 25 non-blood-fed females (2–4 days of age) were introduced into WHO tubes containing impregnated filter papers and exposed for 60 min. For every test, a control assay was run in parallel with untreated papers. The number of knockdown (Kd) mosquitoes was counted every 5 min during the 60 min. After exposure, females were transferred for 24 h into holding tubes (and allowed to feed on a 10% sugar solution) before being checked for mortality. Assays were carried out at 28 ± 2 °C and a relative humidity of 80 ± 10%. The susceptibility of the mosquitoes was classified according to the WHO criteria [45] as susceptible when mortality was > 97%, resistant when mortality was < 90% and possibly resistant when mortality was 90–97%. Surviving females were killed and stored individually at -80 °C in Eppendorf tubes (according to insecticide); dead mosquitoes were stored in silica gel. As in the larval bioassays, Win DL software version 2.0 was used to estimate the KdT_50_ and KdT_95_ and their 95% CIs.

### Screening of *kdr* mutation in *Aedes aegypti* and *Aedes albopictus*

DDT- and deltamethrin-resistant specimens of *Ae. aegypti* and *Ae. albopictus* were examined for the presence of *kdr* mutations. The total DNA of each surviving specimen was extracted with the 2% cetyl trimethyl ammonium bromide protocol described by Morlais et al. [46], then resuspended in 1:50 μl sterile water and stored at −20 °C. The sodium channel gene was examined by PCR and direct sequencing of fragment encoding to verify the presence of mutations at *I1011M* or *I1011V*, *V1016G* or *V1016I* and *F1534C* in *Ae. aegypti* and *F1534C* in *Ae. albopictus*. In *Ae. aegypti*, we amplified and sequenced a part of segment 6 of domain II with the primers AaSCF1 and AaSCR4 [35] and domain III with AaSCF7 and AaSCR7 [35]. Whereas, *Ae. albopictus* was amplified with AaSCF7 and AaSCR7 [37] for domain III.

The PCR mixture contained 2.5 μl 10× buffer (Eurogentec, USA), 1.5 MgCl_2_, 1 mM dNTP (Eurogentec, USA), 1 μl of 1/10 of each primer, 0.1 μl of Diamond *Taq* DNA polymerase (Eurogentec, USA) and 4 μl of 1/50 diluted total DNA in a total volume of 25 μl. DNA was amplified in a GeneAmp 9600 thermal cycler (PerkinElmer, USA) under the following conditions: 94 °C for 3 min, followed by 40 cycles of 30 s at 94 °C, 45 s at the annealing temperature 64 °C, 45 s at 72 °C and 10 min at 72 °C. PCR products (10 μl) were migrated on 1.5% agarose gel in TAE buffer, purified with AMPure® Agencourt® (Beckman Coulter, Danvers MA, USA) when they contained amplified fragments of the expected size and then sequenced with a BigDye Terminator v3.1 Cycle sequencing kit (Applied Biosystems, USA). The primers used for sequencing were: AeSCF3 for *I1011M/V*, AeSCR6 for *V1016G/I* and AeSCR8 for *F1534C* mutations in *Ae. aegypti* and AeSCR8 and AeSCR7 for the *F1534C* mutation in *Ae. albopictus*.

The sequence reaction was carried out in 9 μl of reaction volume containing 1 μl of 5× buffer sequence, 1 μl of primer (5 pmol), 1 μl of Big Dye terminator V3.1 and 1 μl of purified amplicons. Amplification was performed at 96 °C for 1 min, followed by 25 cycles at 96 °C for 10 s, 50 °C for 5 s and 60 °C for 4 min. Direct DNA sequencing was performed on an automatic sequencer ABI 3130XL (Applied BioSystems). Sequences were analysed with BioEdit version 7.2.5 [47].

### Biochemical assays

In order to detect metabolic resistance, biochemical analyses were performed on 2-3-day-old F_0_ mosquito females by the modified microplate methods described by Hemingway & Karunaratne [48] and Brogdon et al. [49]. None of the mosquitoes used had been exposed to insecticide. For each experiment, at least 25 mosquitoes per species were assayed for MFO, GST, acetylcholinesterase (AChE), α- and β-esterases and total proteins. The SBE strain was used as the susceptible reference. Two wells filled with 10 μl of sterile distilled water were used as background (in two adjacent wells) for each analysis. Mosquitoes were ground individually on ice in 200 μl of sterile distilled water. Before centrifugation, 25 μl of each homogenate were distributed in two adjacent wells of a microplate to test for acetylcholinesterase, and the rest of the homogenate was centrifuged at 14,000 rpm for 2 min. Total protein and other enzyme systems were analysed in two replicates. The volume of supernatant was 20 μl for MFO and 10 μl for GST, α- and β-esterases and total proteins. Absorbance was measured in a spectrophotometer for microplate readers (TECAN, Sunrise™) with Magellan version 7.0. software.

The ratios of the specific activities of enzymes to the protein content of individual homogenates were determined in the Bradford assay [50]. Thus, 10 μl of each homogenate was mixed in a microtitre plate well with 140 μl of Bradford reagent (Sigma-Aldrich, St Louis, USA), and the mixture was incubated at room temperature for 5 min. The end-point absorbance was read at 595 nm. Protein values were calculated from a standard curve of absorbance of bovine serum albumin.

### Mixed function oxidase assay

The assay used to measure mixed-function oxidases detects increases in the amount of haem, which are then converted into equivalent units of cytochrome P450. Cytochrome P450 was titrated in the haem-peroxidase assay according to Brogdon et al. [49]. Briefly, 80 μl of 0.625 M potassium phosphate buffer (pH 7.2) were added to 20 μl of microfuged supernatant and 200 μl of tetramethylbenzidine solution (0.011 g 3,3′,5,5′-tetramethylbenzidine + 5 ml methanol + 15 ml sodium acetate buffer 0.25 M, pH 5.0). After addition of 25 μl of 3% hydrogen peroxide, the mixture was incubated for 15 min at room temperature with a cover. Absorbance was read at 630 nm and values calculated from a standard curve.

### *Glutathione* S*-transferase assay*

Glutathione *S*-transferase was measured with a 200 μl 1-chloro-2,4-dinitrobenzene working solution (100 μl of 0.060 g reduced glutathione prepared in 10 ml 0.1 M sodium phosphate buffer pH 6.5 + 100 μl of 0.013 g 63 mM 1-chloro-2,4-dinitrobenzene, diluted in 1 ml methanol and 10 ml 0.1 M phosphate buffer at pH 6.5) added to each replicate of mosquito homogenate. Absorbance was read at 340 nm every 30 s for 5 min. The rate of formation of conjugated 1-chloro-2,4-dinitrobenzene was assessed kinetically from a molecular extinction coefficient (9.5 mM.cm^-1^).

### *Acetylcholinesterase assay*

Two 25-μl replicates from each mosquito homogenate were placed in adjacent wells of a microtitre plate. Membrane-bound acetylcholinesterase in the mosquito homogenate was solubilized by adding 145 μl of Triton phosphate buffer (1% (v/v) Triton X-100 in 0.1 M phosphate buffer, pH 7.8). To one set of homogenates, 25 μl of 0.01 M acetylthiocholine iodide (ASChI) and 10 μl of 0.1 M propoxur solution (2.5 ml 0.1 M ASChI + 10 μl of 0.1 M propoxur in acetone) were added. To the other replicate, 25 μl of ASChI alone was added. The preparation was incubated for 3 min at room temperature, and the kinetics of the enzyme reaction was monitored continuously at 420 nm for 5 min. The results were expressed as percentage remaining activity in the inhibited fraction and in the control (uninhibited).

### *Esterase assays*

Non-specific esterase activity was assessed with two substrates, α- and β-naphthol acetate. For both substrates, 90 μl of phosphate buffer saline (pH 6.5) containing 1% Triton were added to the plate well containing 10 μl of microfuge supernatant, and the reactions were incubated at ambient temperature for 10 min, when 100 μl 0.6 M naphthol was added. After 30 min incubation at ambient temperature, 100 μl of Fast Garnett BC solution (0.010 g Fast Garnett salt + 12 ml distilled water) were added to stop the reaction. The reaction was then incubated for 10 min at room temperature. The concentration of the final product was determined at 550 nm calculated from a standard curve of α- or β-naphthol. Esterase-specific activity per individual was reported as μmol product.min^-1^.mg^-1^ protein.

### Biochemical data analysis

Mean absorbance values of replicate wells for each tested mosquito were converted into enzyme activity and divided by the protein values. The mean activity of each sample was calculated and the distributions of enzymatic activities from wild mosquitoes were compared to those of reference strain mosquitoes (*Ae. aegypti* SBE) using Mann-Whitney tests (nonparametric) with GraphPad Prism version 5.00 software and Mann-Withney *U-test* with MedCalc version 15. Statistical significance was assumed at *P* < 0.05. Mann-Whitney test or Kruskal-Wallis test as appropriated was performed with STATA/IC version 11 (StataCorp College Station, Texas 77845) to assess the association between resistance status and enzymes activities in wild specimens but also to the reference strain, and *P-*values < 0.05 were considered statistically significant.

## Results

### Susceptibility to larvicides

*Aedes aegypti* and *Ae. albopictus* larvae from all sites were susceptible to both larvicides, *Bti* and temephos (Tables 1 and 2). For *Ae. aegypti*, the LC_50_ of *Bti* was 0.06–0.15 mg/l and the LC_95_ 0.25–0.30 mg/l, with similar results in *Ae. albopictus* (LC_50_, 0.07–0.11 mg/l and LC_95_, 0.20–0.27 mg/l). For temephos, the LC_50_ in *Ae. aegypti* was 0.0049–0.0059 mg/l and the LC_95_ 0.0076–0.0096 mg/l, with values in *Ae. albopictus* of 0.0042–0.0057 mg/l and 0.0066–0.01 mg/l, respectively.

**Table 1:** Larval bioassays with *Bacillus thuringensis israelensis* against *Aedes aegypti* and *Ae. albopictus* larvae

Strain and site	*n*	LC_50_ (mg/l) (95% CI)	LC_95_ (mg/l) (95% CI)	RR_50_	RR_95_
*Ae. aegypti*					
IPB	500	0.06 (0.05–0.08)	0.27 (0.22–0.38)	0.85	1.58
Sica 1	500	0.07 (0.04–0.09)	0.25 (0.20–0.35)	1.00	1.47
Lakouanga	500	0.15 (0.12–0.16)	0.30 (0.26–0.42)	1.85	1.57
Ouango	498	0.06 (0.05–0.09)	0.25 (0.20–0.37)	0.85	1.47
Ngongonon 3	500	0.08 (0.06–0.10)	0.27 (0.22–0.36)	1.14	1.58
92 Logements	na	–	–		
*Ae. albopictus*					
IPB	500	0.09 (0.06–0.12)	0.21 (0.18–0.23)	1.28	1.23
Sica 1	500	0.08 (0.05–0.10)	0.27 (0.22–0.37)	1.14	1.58
Lakouanga	500	0.11 (0.07–0.16)	0.26 (0.21–0.32)	1.57	1.52
Ouango	500	0.07 (0.05–0.09)	0.23 (0.19–0.30)	1.00	1.35
Ngongonon 3	500	0.10 (0.07–0.11)	0.2 (0.20–0.27)	1.42	1.17
92 Logements	na	–	–		
Reference strain	500	0.07 (0.05–0.08)	0.17 (0.15–0.21)		

*Abbreviations*: *na* not available; LC_50_ and LC_95_, 50 and 95% lethal concentrations; *CI* Confidence interval, *RR* Resistance ratio

**Table 2:** Larval bioassays with temephos against *Aedes aegypti* and *Ae. albopictus* larvae

Strain and site	*n*	LC_50_ (mg/l) (95% CI)	LC_95_ (mg/l) (95% CI)	RR_50_	RR_95_
*Ae. aegypti*					
IPB	800	0.0055 (0.0047–0.0060)	0.0089 (0.0084–0.0098)	1.17	1.21
Sica 1	800	0.0059 (0.0052–0.0064)	0.0096 (0.0089–0.0108)	1.25	1.31
Lakouanga	800	0.0054 (0.0043–0.0063)	0.0076 (0.0068–0.0082)	1.14	1.04
Ouango	790	0.0052 (0.0046–0.0061)	0.0082 (0.0076–0.0091)	1.10	1.12
Ngongonon 3	788	0.0049 (0.0035–0.0057)	0.0079 (0.0073–0.0085)	1.08	1.08
92 Logements	782	0.0057 (0.0038–0.0061)	00095 (0.0080–0.0093)	1.21	1.30
*Ae. albopictus*					
IPB	800	0.0050 (0.0014–0.0063)	0.0091 (0.0083–0.0148)	1.06	1.24
Sica 1	800	0.0050 (0.0012–0.0068)	0.01 (0.078–0.0127)	1.06	1.36
Lakouanga	797	0.0055 (0.0047–0063)	0.0082 (0.0077–0.0133)	1.17	1.12
Ouango	769	0.0048 (0.0037–0.0058)	0.0078 (0.0067–0.0084)	1.02	1.06
Ngongonon 3	800	0.0057 (0.0046–0.0063)	0.0098 (0.0091–0.011)	1.21	1.34
92 Logements	788	0.0042 (0.0034–0.0054)	0.0066 (0.0055–0.0078)	0.89	0.90
Reference strain	800	0.0047 (0.0020–0.0108)	0.0073 (0.0055–0.0097)		

*Abbreviations*: LC_50_ and LC_95_, 50 and 95% lethal concentrations; *CI* Confidence interval, *RR* Resistance ratio

### Susceptibility to adulticides

The results of WHO tube tests on *Ae. aegypti* and *Ae. albopictus* collected in six districts of Bangui are presented in Table 3. The mortality rate of negative controls was < 5%. In *Ae. aegypti*, resistance to DDT was observed in four populations, with mortality rates of 71–80%, and probable resistance in two further populations, with mortality rates of 93 and 94%. Only one population of *Ae. albopictus* was susceptible (100% mortality) and the rest was either suspected to be resistant (95–97% mortality) or resistant (41–90% mortality). With deltamethrin, *Ae. aegypti* from three localities showed mortality rates ≥ 98%, while the rates in other populations were between 95 and 97%. Samples of *Ae. albopictus* collected at two sites were resistant (rates of 87 and 94%), while all other populations were sensitive. Bioassays with propoxur and fenitrothion showed full susceptibility of the populations of both species, except for one population of *Ae. albopictus* to propoxur (mortality rate of 94%) and one of *Ae. albopictus* to fenitrothion (mortality rate of 96.4%).

**Table 3:** Mortality rates of adult *Aedes aegypti* and *Ae. albopictus* from Bangui and neighbourhoods 24 h after exposure to insecticides

Species and site	% mortality (no. of mosquitoes assayed)							
	Organochlorine		Pyrethroid		Carbamate		Organophosphate	
	4% DDT	Status	0.05% deltamethrin	Status	0.1% propoxur	Status	0.5% fenitrothion	Status
*Ae. aegypti*								
IPB	71.5 (88)	R	95.4 (88)	RS	99 (100)	S	100 (88)	S
Sica 1	78 (100)	R	95.6 (93)	RS	100 (100)	S	100 (100)	S
Lakouanga	79.5 (93)	R	98 (100)	S	100 (100)	S	100 (100)	S
Ouango	93.4 (92)	RS	100 (50)	S	98 (50)	S	100 (100)	S
Ngongonon 3	94 (100)	RS	97 (100)	RS	100 (99)	S	100 (88)	S
92 Logements	78 (50)	R	100 (100)	S	100 (50)	S	na	–
*Ae. albopictus*								
IPB	96.2 (79)	RS	100 (100)	S	100 (75)	S	100 (89)	S
Sica 1	80.2 (96)	R	87 (100)	RS	99 (98)	S	100 (93)	S
Lakouanga	95.7 (71)	RS	99 (89)	S	100 (94)	S	100 (100)	S
Ouango	90 (97)	R	99 (97)	S	100 (100)	S	100 (100)	S
Ngongonon 3	100 (80)	S	100 (93)	S	100 (84)	S	96.4 (84)	RS
92 Logements	41 (100)	R	94 (100)	RS	94 (50)	RS	100 (100)	S
Reference strain	100 (100)		100 (100)		100 (100)		100 (100)	

*Abbreviations*: *na* Not available, *R* Resistant, *RS* Suspected resistance, *S* Susceptible

### Knockdown time

The KDT_50_ calculated after exposure to DDT ranged from 35 to 80 min for *Ae. aegypti* and from 31 to 81.4 min for *Ae. albopictus* (Table 4). The resistance ratio at KDT_50_ for DDT was increased for populations of both species considered as resistant based on their mortality rates. However, these increases were rather limited and ratios did not exceed 1.6. With deltamethrin, all the KDT_50_ values for both species were < 30 min, and the resistance ratio at KDT_50_ was also increased for some populations considered as possibly resistant but ratios were low and below 2.

**Table 4:** Knockdown times in minutes of *Aedes aegypti* and *Ae. albopictus* exposed to 4% DDT and 0.05% deltamethrin. Estimates are from probit analyses with 95% confidence intervals (CI). Resistance ratios (RRs) are calculated as the ratio of the KDT_**50**_ of the field population to that of the control population

Insecticide	Site	*Ae. aegypti*			*Ae. albopictus*		
		KDT_50_ (95% CI)	KDT_95_ (95% CI)	KDT_50_ ratio (RR)	KDT_50_ (95% CI)	KDT_95_ (95% I CI)	KDT_50_ ratio (RR)
4% DDT							
	IPB	63 (57.6–71.9)	115.6 (100.7–139.2)	1.2	46.5 (43.2–50.7)	91.4 (82.1–104.8)	1
	Sica 1	55.5 (53.4–58.1)	85.2 (80.0–92.1)	1.1	81.4 (70.0–103.5)	133.0 (109.1–180.7)	1.6
	Lakouanga	61.2 (57.9–65.5)	98.0 (90.1–108.8)	1.2	48.0 (46.1–50.3)	71.4 (67.0–77.3)	1
	Ouango	34.6 (32.6–36.5)	61.1 (57.4–65.7)	0.7	76.6 (65.1–98.0)	172.0 (138.0–237.5)	1.5
	Ngongonon 3	40.4 (39.3–41.6)	60.1 (57.9–62.6)	0.8	30.5 (27.8–33.3)	57.4 (52.2–64.8)	0.6
	92 Logements	79.8 (69.9–98.5)	127.6 (106.3–169.5)	1.6	65.5 (60.6–78.8)	80.7 (71.1–108.1)	1.3
	Reference strain	50.2 (47.3–52.2)	90.4 (85.3–94.)	1	–	–	
0.05% deltamethrin							
	IPB	14.8(11.9–15.2)	23.5 (21.3–25.8)	1.1	15.8 (8.1–21.5)	27.8 (21.9–48.7)	1.2
	Sica 1	21.2 (14.9–26.3)	40 (34.2–54.5)	1.6	26.8 (18.2–33.6)	54.9 (45.4–77.2)	2
	Lakouanga	12.1 (10.7–14.7)	27.4 (24.8–29.7)	1	14.3 (11.8–16.1)	23.5 (18–38.1)	1.1
	Ouango	23.1(20.3–25.7)	42.01 (38.0–47.7)	1.7	10.1 (4.4–13.6)	24.0 (19.5–34.3)	0.8
	Ngongonon 3	14.1 (12.1–15.9)	26.3 (23.6–0.4)	1.1	13.9 (12.5–15.2)	28.1 (26.0–31.0)	1
	92 Logements	18.6 (17.8–19.5)	29.3 (27.9–30.9)	1.4	23.6 (13.8–30.9)	45.0 (36.4–67.7)	1.8
	Reference strain	13.4 (10.2–14.6)	24.8 (22.3–24.1)	1	–	–	

### Genotyping of *kdr* mutations

We analysed 80 *Ae. aegypti* and 93 *Ae. albopictus* specimens that were survivors of the adult bioassays. None of the non-synonymous mutations in the voltage-gated sensitive channel was detected.

### Biochemical assays

**Fig. 2:**
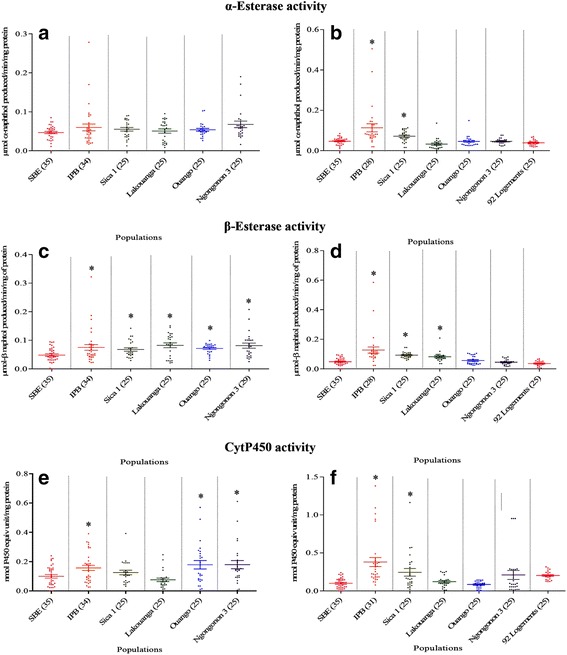
Activity profiles of non-specific α- and β-esterases and mixed-function oxidases (cytochrome P450) in *Ae. aegypti* (**a**, **c**, **e**) and *Ae. albopictus* (**b**, **d**, **f**). Numbers in parentheses indicate the number of mosquitoes assayed. Asterisks indicate significant increase in wild populations compared to SBE susceptible strain values (*P* < 0.05, Mann-Whitney tests). *Abbreviations*: SBE, Benin strain; IPB, Institut Pasteur de Bangui; NES, non-specific esterase; MFO, mixed-function oxidase

**Fig. 3:**
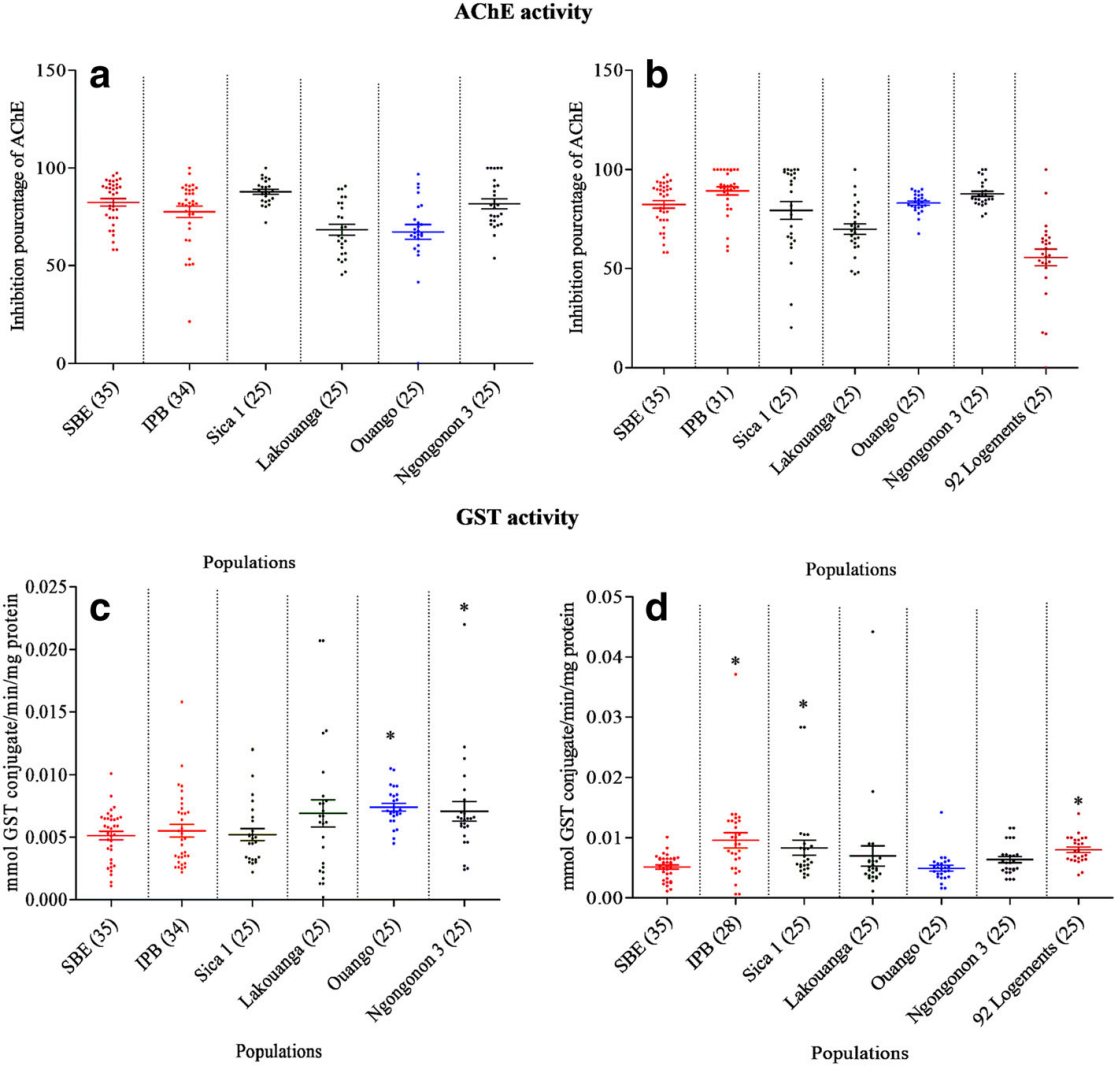
Profiles of acetylcholinesterase inhibition and glutathione *S*-transferase in *Ae. aegypti* (**a**, **c**) and *Ae. albopictus* (**b**, **d**). Numbers in parentheses indicate the number of mosquitoes assayed. Asterisks indicate significant differences compared to SBE susceptible strain values (*P* < 0.05, Mann-Whitney tests). *Abbreviations*: SBE, Benin strain; IPB, Institut Pasteur de Bangui; AChE, acetylcholinesterase; GST, glutathione *S*-transferase

For each of the four enzyme systems tested, we analysed at least 25 individuals per species and per population (Figs. 2 and 3). Comparisons with the SBE strain showed significant differences (Mann-Whitney *U-test*, *P* < 0.05) in mean enzyme activity in some populations: α-esterase activities were significantly higher in two *Ae. albopictus* populations (IPB, *U* = 140, *Z* = 4.84, *P* < 0.0001, and Sica 1, *U* = 216, *Z* = 3.32, *P* = 0.0009), and β-esterase activities were significantly higher in all five populations of *Ae. aegypti* (IPB,*U* = 429.5, *Z* = 1.98, *P* = 0.04; Sica 1, *U* = 284, *Z* = 2.3, *P* = 0*.*02; Lakouanga, *U* = 258, *Z* = 2.69, *P =* 0.007; Ouango, *U* = 214.5, *Z* = 3.34, *P* = 0.0008; and Ngongonon 3, *U* = 230.5, *Z* = 3.1, *P =* 0.001) and three *Ae. albopictus* populations (IPB, *U* = 118, *Z* = 5.14, *P* < 0.0001; Sica 1, *U* = 84, *Z* = 5.3, *P* < 0.0001; and Lakouanga, *U* = 168, *Z* = 4.04, *P* = 0.0001.

Significantly higher MFO activity was observed in three *Ae. aegypti* populations (IPB, *U* = 404, *Z* = 2.29, *P* = 0.02; Ouango, *U* = 296, *Z* = 2.12, *P* = 0.03; and Ngongonon 3, *U* = 296.5, *Z* = 2.11, *P* = 0.03) and two *Ae. albopictus* populations (IPB, *U* = 66, *Z* = 5.86, *P* < 0.0001 and Sica 1, *U* = 264, *Z* = 2.6, *P* = 0.009). The mean rate of inhibition of AChE activity in the presence of propoxur was significantly lower in two *Ae. aegypti* populations (Lakouanga, *U* = 191, *Z* = 3.69, *P* = 0.0002 and Ouango, *U* = 206, *Z* = 3.47, *P* = 0.0005) and in two *Ae. albopictus* populations (Lakouanga, *U* = 205, *Z* = 3.48, *P* = 0.0005 and 92 Logements, *U* = 92, *Z* = 5.18, *P* < 0.0001), suggesting that they contained some individuals with reduced sensitivity. Glutathione *S*-transferase activity was significantly higher than control in two populations of *Ae. aegypti* (Ouango, *U* = 155.5, *Z* = 4.22, *P* < 0.0001 and Ngongonon 3,*U* = 295, *Z* = 2.13, *P* = 0.03) and in three populations of *Ae. albopictus* (IPB, *U* = 223, *Z* = 3.69, *P* = 0.0002, Sica 1, *U* = 287, *Z* = 2.25, *P* = 0.024 and 92 Logements, *U* = 156.5, *Z* = 4.24, *P* < 0.0001). Statistical analysis performed to assess association between enzyme activities and resistance status of *Aedes* spp. populations. This showed that resistance status is significantly associated to certain enzyme activities such as GST and CytP450 in *Ae. aegypti* (Additional file 1: Table S1), and EST (α- and β-), GST and CytP450 in *Ae. albopictus* (Additional file 1: Table S2). This showed also that enzyme activities of field strain in both *Aedes* spp. is generally high than those of susceptible laboratory strains.

## Discussion

The results of the larval bioassays with *Bti* and temephos indicated satisfactory susceptibility in all samples of *Ae. aegypti* and *Ae. albopictus* assayed. Full resistance to *Bti* has not been reported in field populations of either *Aedes* species although it was found in *Culex pipiens* in New York State [51]. This larvicide acts as a mixture of toxins with different modes of action, which reduces resistance in targeted populations. As *Bti* is highly specific to some Diptera and might be considered a biological control agent, it is the first choice of larvicide for use against *Aedes* species [39, 52]. For temephos, our results suggest full susceptibility of all the tested populations. Nevertheless, resistance to this compound has been reported in *Ae. aegypti* in Brazil [53] and Santiago Island in Cape Verde [54] and in *Ae. albopictus* in Greece [55]. Insecticide resistance results from extensive, long-term use [20, 21]; thus, for example, in Brazil, resistance to temephos compromises its use by the national dengue control programme [56, 57]. Although this insecticide was used moderately in Central Africa (Cameroon and Gabon) in the 1970s [38], it had never been used in vector control programmes in CAR, which probably explains the full susceptibility we observed for both species.

None of the *Ae. aegypti* populations from Bangui can be considered fully susceptible to DDT, as all were either possibly resistant or resistant. The same was true for *Ae. albopictus*, although the mortality rates were sometimes lower (41%). Mouchet et al. [38] previously reported decreased susceptibility to DDT in *Ae. aegypti* sampled in Bangui in 1965 and 1971, suggesting continuing selection pressure on *Aedes* populations. DDT resistance has repeatedly been reported in *Ae. aegypti* [24, 58] and *Ae. albopictus* [59, 60], although data for the latter are scarce. The decreased susceptibility to deltamethrin observed in both populations may represent an obstacle for vector control programmes, because pyrethroids are recommended for the control of adult *Aedes* mosquitoes [61, 62].

The resistance to DDT and deltamethrin observed in both species is difficult to explain, because in CAR, as in other countries of Central Africa, use of insecticides against *Ae. aegypti* and *Ae. albopictus* is limited [39]. CAR has promoted use of long-lasting insecticidal nets as one of the main components of the national malaria control programme, with indoor residual spraying by households with insecticides available on the market, which we noted at nearly every study site. Therefore, insecticides used against other insects of medical or agricultural importance may exert indirect selection pressure on these two mosquito species [63, 64]. Similar observations have been made for resistance of *Ae. aegypti* to deltamethrin in Indonesia [65], Thailand [61] and Nigeria [58]. As *Ae. albopictus* was reported for the first time in CAR in 2009 [66], we cannot exclude the possibility of invading populations with resistance to DDT, as suggested by Kamgang et al. [39] in Cameroon.

In this study, the *kdr* mutations *I1011M/V*, *V1016G/I*, *F1534C* in *Ae. aegypti* and F1534C in *Ae. albopictus* were not detected in mosquitoes that survived to DDT or deltamethrin, in accordance with the finding that knockdown time was not or weakly increased during insecticide exposure in test tubes. We observed increased activity of several enzymes in *Ae. aegypti* and *Ae. albopictus* samples that might explain the decreased susceptibility to DDT and pyrethroids. Although the mean activity of β-esterase was increased significantly in some populations of *Ae. aegypti* and *Ae. albopictus*, there was no evidence of resistance to deltamethrin in these samples according to WHO diagnostic concentrations [42]; however, most of the WHO diagnostic concentrations were established for *Anopheles* species, and it is possible that they are not transposable to *Aedes* or *Culex* mosquitoes. The diagnostic concentrations on these species for the main insecticides used in control vector should therefore be assessed. Although elevated esterase and glutathione *S*-transferase activities can be involved in temephos resistance [67], in our study all the populations were fully susceptible to this insecticide.

High MFO activity was found in three *Ae. aegypti* and three *Ae. albopictus* populations, but also with no clear association with full resistance to deltamethrin. In contrast, Paeporn et al. [68] in Thailand showed that increased MFO and esterase activities in *Ae. aegypti* strains were associated with pyrethroid (deltamethrin and permethrin) resistance, and Saavedra-Rodriguez et al. [69] reported that mixed-function oxidases and esterases were important in resistance to organophosphate insecticides in *Ae. aegypti* populations in Latin America. Reduced activity of AChE to propoxur inhibition was detected in some individuals of *Ae. aegypti* and *Ae. albopictus*, suggesting possible emergence of resistance to carbamates or other organophosphates in field populations.

## Conclusion

We describe for the first time the susceptibility of *Aedes* in CAR to the main insecticide classes and the mechanisms potentially involved in resistance. This information adds to that on the susceptibility of *Ae. aegypti* and *Ae. albopictus* to commonly used insecticides in Central Africa. The susceptibility of both species to *Bti* and temephos is encouraging for larval control in Bangui; however, most *Ae. aegypti* and *Ae. albopictus* samples were resistant or suspected of being resistant to DDT. Moreover, some populations of both species showed possible resistance to deltamethrin according to the WHO criteria. The absence of *kdr* mutations in the two species cannot explain the decreased sensitivity; however, differences in the activity of certain enzymes involved in metabolism could explain differences in susceptibility between populations. The results with propoxur (carbamate) and fenitrothion (organophosphate) were satisfactory, as decreased sensitivity was found in only one population of *Ae. albopictus* for propoxur and one for fenitrothion. These findings are important for effective control of DENV, CHIKV and ZIKV vectors in CAR. Further studies with the Centers for Disease Control and Prevention bottle bioassay and synergists should be conducted to obtain additional information on metabolic-mediated resistance mechanisms.

## Additional files

Additional file 1: Table S1. Assessing association between resistance status and enzyme activities in *Ae. aegypti.* **Table S2.** Assessing association between resistance status and enzyme activities in *Ae. abopictus. (DOC 135 kb)*

## Abbreviations

ASChI: Acetylthiocholine iodide
*Bti*: *Bacillus thuringiensis* var *israeliensis*
CAR: Central African Republic
CHIKV: Virus of chikungunya
CIs: Confidence intervals
DDT: Dichlorodiphenyltrichloroethane
DENV: Virus of dengue
DNA: Deoxyribonucleic acid
dNTP: Deoxyribonucleotide triphosphate
GST: Glutathione *S*-transferase
IPB: Institut Pasteur de Bangui
Kd: Knockdown
Kdr: Knockdown resistance
KDT: Knockdown time
LC: Lethal concentration
MFO: Mixed-function oxidase
NA: Not available
NES: Non-specific esterase
PCR: Polymerase chain reaction
rpm: Rotations (or revolutions) per minute
RR: resistance ratio
SBE: Strain from Benin
TAE: Tris-acetate-EDTA
WHO: World Health Organization
