# Supplementary material for: Susceptibility profile and metabolic mechanisms involved in Aedes aegypti and Aedes albopictus resistant to DDT and deltamethrin in the Central African Republic
Source: Parasit Vectors. 2016 Nov 24;9:599. doi: 10.1186/s13071-016-1887-5 (PMC5121976; doi:10.1186/s13071-016-1887-5)
Supplement: Additional file 1: Table S1. — Assessing association between resistance status and enzyme activities in Ae. aegypti. Table S2. Assessing association between resistance status and enzyme activities in Ae. abopictus. (DOC 135 kb) [file 13071_2016_1887_MOESM1_ESM.doc]

**Additional file 1. Table S1.** Assessing association between resistance status and enzyme activities in *Ae. aegypti*

|  | **Insecticide** | **Enzyme** | **Status** | **Means of enzymatic activity** | **P ^(a)^** | **P ^(b)^** |
| --- | --- | --- | --- | --- | --- | --- |
|  | DDT | α- esterase | R | 0.0557 | 0.3575 | 0.4113 |
|  |  |  | RS | 0.0606 |  | 0.0876 |
|  |  | β-esterase | R | 0.0750 | 0.3734 | 0.0040* |
|  |  |  | RS | 00.763 |  | 0.0001* |
|  |  | GST | R | 0.0058 | 0.0150* | 0.7621 |
|  |  |  | RS | 0.0072 |  | 0.0473* |
|  |  | CytP450 | R | 0.1236 | 0.0009* | 0.6759 |
|  |  |  | RS | 0.1789 |  | 0.0003* |
|  | Deltamethrin | α- esterase | RS | 0.0607 | 0.6637 | 0.2059 |
|  |  |  | S | 0.0521 |  | 0.3091 |
|  |  | β-esterase | RS | 0.0747 | 0.1837 | 0.0024* |
|  |  |  | S | 0.0768 |  |  |
|  |  | GST | RS | 0.0059 | 0.0125* | 0.4216 |
| ***Ae. aegypti*** |  |  | S | 0.0071 |  | 0.0028* |
|  |  | CytP450 | RS | 0.1544 | 0.0260* | 0.0996 |
|  |  |  | S | 0.1273 |  | 0.9105 |
|  | Propoxur ^(NA)^ | α- esterase | S | NA | NA | NA |
|  |  | β-esterase | S | NA | NA | NA |
|  |  | GST | S | NA | NA | NA |
|  |  | CytP450 | S | NA | NA | NA |
|  | Fenitrothion ^(NA)^ | α- esterase | S | NA | NA | NA |
|  |  | β-esterase | S | NA | NA | NA |
|  |  | GST | S | NA | NA | NA |
|  |  | CytP450 | S | NA | NA | NA |

^(a)^: Comparison of enzyme activities between wild specimens according to the resistance status; ^(b)^: Comparison of enzyme activities of wild specimens for each status to the enzymatic activities for the reference strain ;  ^(NA)^ Not applicable ; *: difference statistically significant; MFO: mixed-function oxidases (cytochrome P450); GST: glutathione *S*-transferase; 1: resistant; 2: resistance suspected; 3: susceptible

**Additional file 1. Table S2.** Assessing association between resistance status and enzyme activities in *Ae. albopictus*

|  | **Insecticide** | **Enzyme** | **Status** | **Means of enzymatic activity** | **P ^(a)^** | **P ^(b)^** |
| --- | --- | --- | --- | --- | --- | --- |
| ***Ae. albopictus*** | DDT | α- esterase | R | 0.0522 | 0.58169 | 0.7805 |
|  |  |  | RS | 0.0751 |  | < 0.0001* |
|  |  |  | S | 0.0463 |  | 0.5539 |
|  |  | β-esterase | R | 0.0614 | < 0.000001* | 0.093 |
|  |  |  | RS | 0.1055 |  | 0.1498 |
|  |  |  | S | 0.0447 |  | 0.6624 |
|  |  | GST | R | 0.0070 | 0.780205 | <0.0001* |
|  |  |  | RS | 0.0083 |  | <0.0001* |
|  |  |  | S | 0.0063 |  | <0.0001* |
|  |  | CytP450 | R | 0.1770 | 0.016221* | <0.0001* |
|  |  |  | RS | 0.2724 |  | < 0.0001* |
|  |  |  | S | 0.2099 |  | < 0.0001* |
|  | Deltamethrin | α- esterase | RS | 0.0552 | 0.5172 | 0,393 |
|  |  |  | S | 0.0611 |  | 0.8972 |
|  |  | β-esterase | RS | 0.0640 | 0.283 | 0.0499* |
|  |  |  | S | 0.0788 |  | 0.0032* |
|  |  | GST | RS | 0.0081 | 0.0049* | < 0.0001* |
|  |  |  | S | 0.0070 |  | < 0.0001* |
|  |  | CytP450 | RS | 0.2231 | 0.0137* | < 0.0001* |
|  |  |  | S | 0.2117 |  | < 0.0001* |
|  | Propoxur | α- esterase | RS | 0.0389 | 0.0087* | 0.0758 |
|  |  |  | S | 0.0631 |  | 0.351 |
|  |  | β-esterase | RS | 0.0356 | < 0.0001* | 0,0877 |
|  |  |  | S | 0.0814 |  | 0.0002* |
|  |  | GST | RS | 0.0079 | 0.0048* | < 0.0001* |
|  |  |  | S | 0.0072 |  | < 0.0001* |
|  |  | CytP450 | RS | 0.2021 | 0.0152* | < 0.0001* |
|  |  |  | S | 0.2180 |  | < 0.0001* |
|  | Fenitrothion | α- esterase | RS | 0.0463 | 0.5085 | 0.5539 |
|  |  |  | S | 0.0617 |  | 0.5276 |
|  |  | β-esterase | RS | 0.0447 | 0.0001* | 0.6624 |
|  |  |  | S | 0.0797 |  | 0.0007* |
|  |  | GST | RS | 0.0063 | 0.5292 | < 0.0001* |
|  |  |  | S | 0.0075 |  | < 0.0001* |
|  |  | CytP450 | RS | 0.2099 | 0.0396* | < 0.0001* |
|  |  |  | S | 0.2165 |  | < 0.0001* |

^(a)^: Comparison of enzyme activities between wild specimens according to the resistance status; ^(b)^: Comparison of enzyme activities of wild specimens for each status to the enzymatic activities for the reference strain ; *: difference statistically significant; MFO: mixed-function oxidases (cytochrome P450); AChE: acetylcholinesterase; GST: glutathione *S*-transferase; R: resistant; RS: resistance suspected; S: susceptible
